# Side‐effects of carbetocin to prevent postpartum hemorrhage: A systematic review and meta‐analysis of randomized controlled trials

**DOI:** 10.1002/prp2.745

**Published:** 2021-03-15

**Authors:** Wen Ai, Yanfei Zeng, Yubo Ma, Li Liu, Dazhi Fan, Song Wu, Yinghui Zhang

**Affiliations:** ^1^ Department of Obstetrics and Gynecology Foshan Chancheng Central Hospital Foshan Guangdong China; ^2^ Department of Epidemiology and Biostatistics School of Public Health Anhui Medical University Hefei Anhui China; ^3^ Department of Library the First Affiliated Hospital College of Medicine Zhejiang University Hangzhou Zhejiang China; ^4^ Foshan Institute of Fetal Medicine Affiliated Foshan Maternity & Child Healthcare Hospital Southern Medical University Foshan Guangdong China; ^5^ School of Integrated Traditional and Western Medicine Anhui University of Chinese Medicine Hefei Anhui China

**Keywords:** carbetocin, meta‐analysis, postpartum hemorrhage, side‐effects, systematic review

## Abstract

Postpartum hemorrhage (PPH) increases the risk of maternal death worldwide. Heat‐stable carbetocin, a long‐acting oxytocin analog, is a newer uterotonic agent. Clinicians do not fully understand its side‐effects, particularly the unanticipated side‐effects. The aim of this study is to investigate the side‐effects of carbetocin to PPH. The Cochrane Library, Web of Science, PubMed, Elsevier ScienceDirect, Embase, and ClinicalTrials.gov were searched from the inception to September 2020. Randomized controlled trials (RCTs) that considered pregnant women who received carbetocin before delivery and provided at least one adverse event were included. Statistical analysis included random or fixed‐effect meta‐analyses using relative risk. Stratified analyses and sensitivity analyses were also performed. Begger's and Egger's test and funnel plots were used to assess the publication bias. Seventeen RCTs involving 32,702 women were included, and all these studies ranked as medium‐ to high‐quality. Twenty‐four side‐effects were reported. The use of carbetocin had a lower risk of vomiting in intravenously (0.53, 0.30 to 0.93) and cesarean birth (0.51, 0.32 to 0.81) women, and had a slightly higher risk of diarrhea (8.00, 1.02 to 62.79) compared with oxytocin intervention. No significant difference was found among other side‐effects. Evidence from our systematic review and meta‐analysis of 17 RCTs suggested that the risk of vomiting decreased with carbetocin use in the prevention of PPH after delivery.

## Graphical abstract

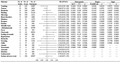

**Abbreviations**
CIs: confidence intervals
PPH: postpartum hemorrhage
RCTs: randomized controlled trials
RR: relative risk

## 1 INTRODUCTION

Postpartum hemorrhage (PPH) caused a significant number of maternal deaths worldwide. About 27.1% of all maternal deaths were caused by hemorrhage, and these data can reach 36.9% in most low‐income countries and regions.^1^ It has already been confirmed that prophylactic administration of uterotonic agents is the most important component in terms of reducing the risk of PPH and preventing the irreversible functional consequences in the stage of labour.^2^

Oxytocin, a short half‐life uterotonic agent, is recommended by the World Health Organization (WHO) as the first line for the prevention and treatment of PPH in 2012.^3^ However, it is sensitive to heat and requires cold storage and transport in usage. The active ingredient and purity are mostly affected in low‐resource settings where the cold chain is not commonly available. Because of its heat sensitivity, it does not possess satisfactory real‐world efficacy, particularly in hot low‐ and middle‐income countries and regions.^4^ Meanwhile, the short half‐life required frequently or continuously repeated administration.

Heat‐stable carbetocin, a long‐acting oxytocin analog, is a newer uterotonic agent. Its effects of uterine contractions can start within two minutes, and the rhythmic contractions can last for 60 to 120 minutes in intravenous and intramuscular injection, respectively.^5^, ^6^ What is important is that it has high thermal stability, and it can be transported and stored at normal temperature and even in hot and humid environments without compromising quality. The heat‐stability data showed that it maintained for a minimum of 36 months at 30°C and 75% relative humidity and at extreme temperatures, such as 50°C, for three months.^7^ Hence, it would be advantageous and even a significant breakthrough for maternal health in hot environments lacking cold chain routes to use carbetocin. Recently, a multicenter clinical trial, including 23 hospitals in 10 countries, indicated that the intramuscular administration of 100 ug of heat‐stable carbetocin was noninferior to the administration of 10 IU oxytocin for the prevention of PPH after vaginal birth.^8^ Meanwhile, systematic reviews and meta‐analysis demonstrated that carbetocin significantly reduced postpartum blood loss, additional uterotonics, and transfusion.^9^, ^10^ The use of carbetocin is recommended for the prevention of PPH for all births by WHO, particularly in settings where oxytocin is unavailable or its quality cannot be guaranteed.^2^

Side‐effects were also an important concern when choosing uterotonic agents. Although carbetocin seems to be an ideal agent compared to other uterotonic agents, some side‐effects, such as vomiting, nausea, and dysarteriotony, are still concerned. There are many trials of carbetocin use to prevent PPH, and side‐effects are always as secondary outcomes in these trials. Clinicians do not fully understand the side‐effects of carbetocin to PPH, particularly the unanticipated ones. There seems to be a gap to detailed presentation of the side‐effects of carbetocin. Therefore, this study aims to assess the side‐effects of prophylactic carbetocin to PPH among the previous randomized clinical trials.

## 2 MATERIALS AND METHODS

This systematic review was pre‐registered online in the PROSPERO registry (CRD42019134522). The perform of the current study followed the Preferred Reporting Items for Systematic Reviews and Meta‐Analyses guidelines. The ethical approval was not required for this study.

### 2.1 Search strategy

A systematic search of the Cochrane Library, Web of Science, PubMed, Elsevier ScienceDirect, ClinicalTrials.gov, and Embase was conducted from the earliest publication date available through May 31, 2019. The search was updated on September 1, 2020. The MeSH search terms including Carbetocin, Postpartum Hemorrhage, and Randomized Clinical Trials were used and were listed in detail in Appendix 1. There is no language restriction. A manually snowball search strategy was also used to identify additional studies from the reference lists of retrieved studies and relevant reviews.

### 2.2 Inclusion and exclusion criteria

Studies were considered if the following criteria were met: 1) randomized controlled trials (RCTs) design, 2) pregnant women received the prophylactic carbetocin before delivery, 3) compared carbetocin with oxytocin or placebo interventions, and 4) provided the frequency of at least one side‐effect. Cluster‐ or quasi‐RCTs, ongoing trials, cross‐sectional studies, case series, abstracts without full text, or studies without sufficient data were excluded.

### 2.3 Study selection and data extraction

Two authors (Wen Ai and Dazhi Fan) independently identified eligible articles on title and abstract first, and then on the full text. The data extraction was also independently performed by the same authors using a prespecified Excel form, and the extracted variables from each study included study and participant characteristics (first author, year of publication, region, trial registration number, funding source, risk of PPH, and mode of delivery), arms and treatment regimens (dose and route), and the type and frequency of side‐effects. Disagreement between authors was solved by discussion between the two authors. Meanwhile, we also entered the data into the EpiDate software to check the accuracy.

### 2.4 Risk of bias assessment

Using the Cochrane Handbook,^11^ two authors (Yanfei Zeng and Yubo Ma) independently assessed the quality of the included studies. Evaluation criteria included the following major domains: randomization, implementation of blind, data reporting, and other bias, such as funding source, and potential conflicts of interest.

### 2.5 Data analyses

The assessment was the relative risk (RR) with 95% confidence intervals (CIs) comparing the frequency of side‐effects between carbetocin and control groups, which was calculated using frequentist pairwise meta‐analysis. Forest plots were used to present the results of RR and 95%CIs. Heterogeneity across studies was assessed using Tau^2^, *I^2^*, and Chi^2^ statistics. The selected effect models, fixed or random, were based on the heterogeneity result.

For all side‐effects, if they were provided by ten or more trials, we stratified analyses by the route of carbetocin administration (intravenously versus intramuscularly), mode of delivery (vaginal versus cesarean birth), prior risk of PPH (high, low or none), trial register (yes or no), funding source (company, researcher, or none), and control‐intervention ways to identify the main sources of heterogeneity between trials. To assess the dose effect, a sensitivity analysis was performed to exclude the trial in which the carbetocin dose was not 100 micrograms. To assess the publication bias, the Begger and Egger tests were used. Data analysis and graphing were conducted using the R software, the Review Manager software, and Microsoft Excel.

## 3 RESULTS

### 3.1 Study identification and characteristics

**FIGURE 1:**
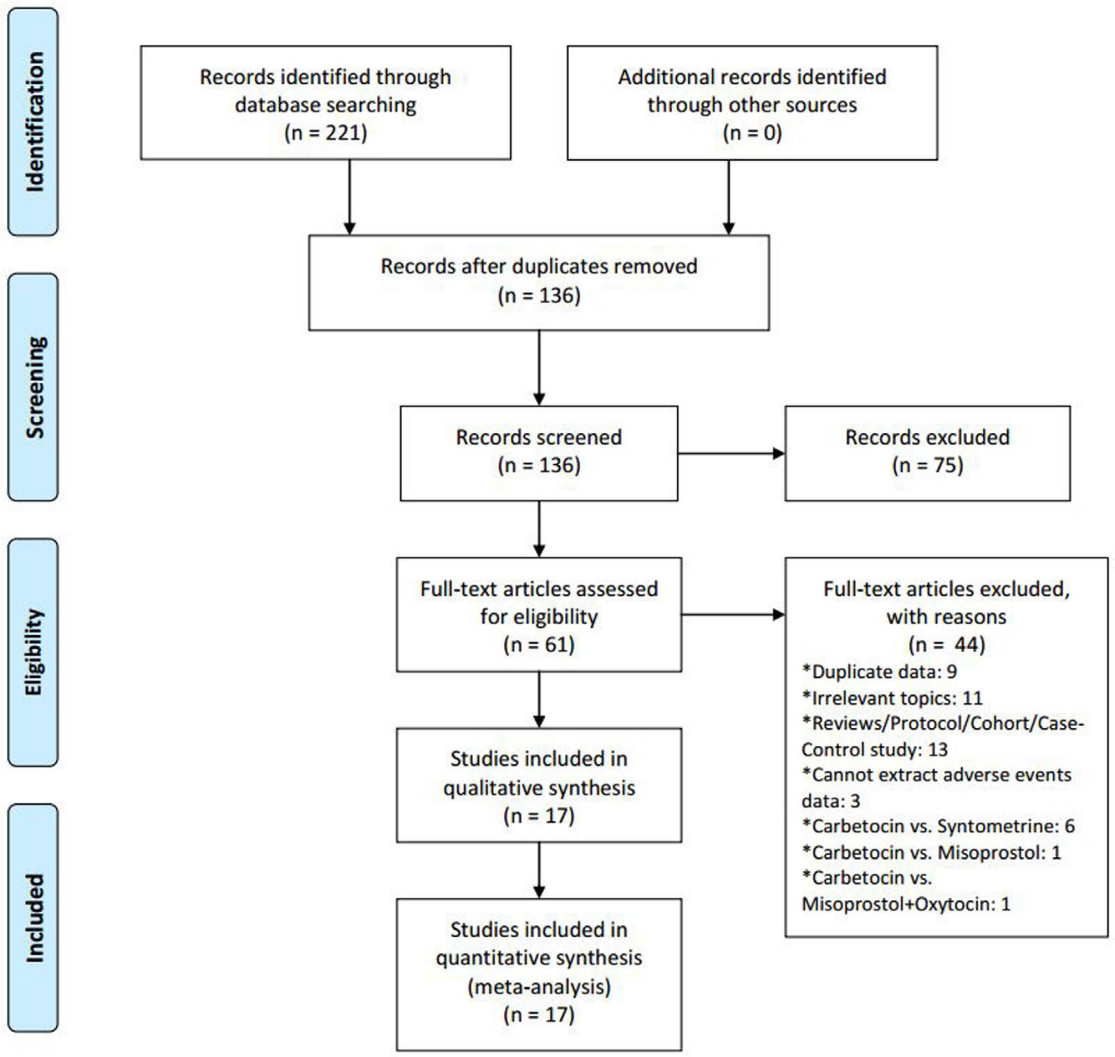
Flow chart of systematic review and meta‐analysis

We first identified 221 potentially eligible articles, and 136 articles were scrutinized for the full text. Ultimately, a total of 17 RCTs^8^, ^27^ involving 32,702 women were included (Figure 1). Two articles^12^, ^13^ contained three arms, respectively. They were published from 1998 to 2018. The median size was 160 participants with the interquartile range of 67 to 299. Of the included 17 studies, 11 articles were designed for women following cesarean deliveries and six were for women undergoing vaginal deliveries. Ten out of 17 articles recruited women with high risk factors for PPH and five recruited women with low risk factors. The risk factor is not specified in two articles.^13^, ^14^ All of the articles compared the use of carbetocin with oxytocin, and only one article contain one trial of carbetocin versus placebo.^12^

Except for Sunil Kumar KS’s article (125 ug),^15^ all articles performed a standard dose of 100 ug carbetocin. The articles were conducted in various countries. The most articles were conducted from the Egypt (n = 3) and Canada (n = 3), followed by Austria, Belgium, India, Iran, Italy, Malaysia, Norway, Panama, Spain, and UK (n = 1 each); and one article^8^ involved 23 hospitals in 10 countries. Eight articles were pre‐registered in the Web‐based registry of clinical trials, such as ClinicalTrials.gov. Four articles were funded by pharmaceutical company, four articles were funded by organizations, university, or hospital, and nine articles did not disclose funding sources. (Table 1).

**TABLE 1:** Characteristics of included studies.

First author	Publish Year	Trial Phase	Trail No.	Funded	Country	Risk for PPH	Delivery Mode	Interventions (sample size; dose; adm)	Side effects
Mannaerts D^16^	2018	NA	ISRCTN 95504420	NA	Belgium	L	CS	Carbetocin (32; 100 ug, i.v.) vs. Oxytocin (26; 20 iu, i.v.)	Nausea Flushing Hypotension Vomiting
Taheripanah R^17^	2018	II	NCT02079558	Shahid Beheshti University of Medical Sciences	Iran	H	CS	Carbetocin (110; 100 ug, i.v.) vs. Oxytocin (110; 30 iu, i.v.)	Vomiting Headache Nausea Tremor Dizziness Pruritus
Widmer M^8^	2018	III	ACTRN12614000870651; 2014–004445–26; CTRI/2016/05/006969	Merch Sharpe & Dohme	Argentina; Egypt; India; Kenya; Nigeria; Singapore; South Africa; Thailand; Uganda; the United Kingdom	L	VD	Carbetocin (14754; 100 ug, i.m.) vs. Oxytocin (14743; 10 iu, i.m.)	Chest pain; Flushing Abdominal pain; Vomiting
El Behery MM^18^	2016	NA	NA	NA	Egypt	H	CS	Carbetocin (90; 100 ug, i.v.) vs. Oxytocin (90; 20 iu, i.v.)	Headache Nausea Vomiting Sweating Palpitation Fever
Maged AM^19^	2016	NA	NA	NA	Egypt	H	VD	Carbetocin (100; 100 ug, i.m.) vs. Oxytocin (100; 100 ug, i.m.)	Nausea Vomiting Tachycardia Flushing Dizziness Headache Shivering Anemia Metallic taste; Dyspnea Palpitations Itching
Maged AM^20^	2016	III	NCT02304055	Cairo University	Egypt	H	VD	Carbetocin (50; 100 ug, i.v.) vs. Oxytocin (50; 5 iu, i.v.)	Nausea Vomiting Tachycardia Flushing Dizziness Headache Shivering Metallic taste; Dyspnea Palpitations Itching
Razali N^21^	2016	NA	ISRCTN18976822	the University of Malaya	Malaysia	L	CS	Carbetocin (276; 100 ug, i.v.) vs. Oxytocin (271; 10 iu, i.v.)	Arrhythmias
Sunil Kumar KS^15^	2016	NA	NA	NA	India	L	VD	Carbetocin (100; 125 ug, i.m.) vs Oxytocin (100; 10 iu, i.m.)	Nausea Vomiting Shivering Diarrhea Fever
Ortiz‐Gomez JR^13^	2013	NA	NA	NA	Spain	NA	CS	Carbetocin (52; 100 ug, i.v.) vs. Oxytocin (52, 52; 1 iu, 20 iu, i.v.)	Vomiting; Nausea; Tremor; Headache; Chest pain;
Rosseland LA^12^	2013	IV	NCT00977769	Ferring Pharmaceutical	Norway	H	CS	Carbetocin (25; 100 ug, i.v.) vs. Oxytocin (26; 5 iu, i.v.) vs. placebo	Metallic taste; Xerostomia Nasal congestion; Headache Flushing Palpitations Shortness of breath; Chest pain Feeling of warmth;
Moertl MG^22^	2011	NA	2007–005498–78; NCT01277978	Medical University of Graz	Austria	H	CS	Carbetocin (28; 100 ug, i.v.) vs. Oxytocin (28; 5 iu, i.v.)	Nausea Flushing Headache Tachycardia Shortness of breath; Feeling warm;
Reyes OA^23^	2011	NA	NA	NA	Panama	H	VD	Carbetocin (26; 100 ug, i.v.) vs. Oxytocin (29; 20 iu, i.v.)	Headaches Palpitations Fever Nausea Vomiting Hot sensation; Flushing Malaise
Attilakos G^24^	2010	NA	2005–002812–94	Ferring UK funded the cost of preparation of the ‘blinded'drug ampoules	UK	High	CS	Carbetocin (188; 100 ug, i.v.) vs. Oxytocin (189; 5 iu, i.v.)	Nausea Vomiting Headache Tachycardia Metallic taste; Backache Abdominal pain; Arm pain; Trigeminy Flushed Shortness of breath; Wheezing Tremors Hypotension Sweating Tightness throat; ST depression; Blurred vision;
Borruto F^25^	2009	NA	NA	NA	Italy	H	CS	Carbetocin (52; 100 ug, i.v.) vs. Oxytocin (52; 10 iu, i.v.)	Anemia Arrhythmias Abdominal pain; Nausea Vomiting Metallic taste; Heat sensation; Back pain; Headache Tremor Dizziness Difficulty in breathing; Dyspnea Chest pain; Pruritus Flushing Hypotension
Boucher M^26^	2004	NA	NA	NA	Canada	H	VD	Carbetocin (83; 100 ug, i.m.) vs. Oxytocin (77; 10 iu, i.v.)	Headache Chills Abdominal pain; Dizziness Tremor Vasodilatation Leukocytosis Nausea Vomiting Pruritis
Dansereau J^27^	1999	NA	NA	A Clinical Research Grant from Ferring Inc., Canada	Canada	H	CS	Carbetocin (329; 100 ug, i.v.) vs. Oxytocin (330; 25 iu, i.v.)	Abdominal pain; Back pain; Headache Nausea Metallic taste; Flushing Sweating Tremors Vomiting Feeling of warmth;
Boucher M^14^	1998	NA	NA	NA	Canada	NA	CS	Carbetocin (29; 100 ug, i.v.) vs. Oxytocin (28; 10 iu, i.v.)	Vomiting; Nausea; Dizziness; Pruritus; Headache; Shortness of breath; Chills

Abbreviations: CS, cesarean section; H, high risk for PPH; HL, high and low risk for PPH; L, low risk for PPH; NA, none; PPH, postpartum hemorrhage; VD, vaginal birth.

A total of 24 side‐effects were reported in this study. Most of articles reported vomiting (14 articles), nausea (14), headache (13), and flushing (10) as side‐effects of carbetocin. Less than ten articles reported side‐effects included shivering (9), heart disorders (9), dizziness (8), dyspnea (7), pruritus (6), metallic taste (6), abdominal pain (5), fever (4), chest pain (4), feeling of warmth (4), hypotension (3), backache (3), sweating (3), chills (2), anemia (2), xerostomia (1), serious adverse event (1), arm pain (1), diarrhea (1), and leukocytosis (1).

### 3.2 Risk of bias

**FIGURE 2:**
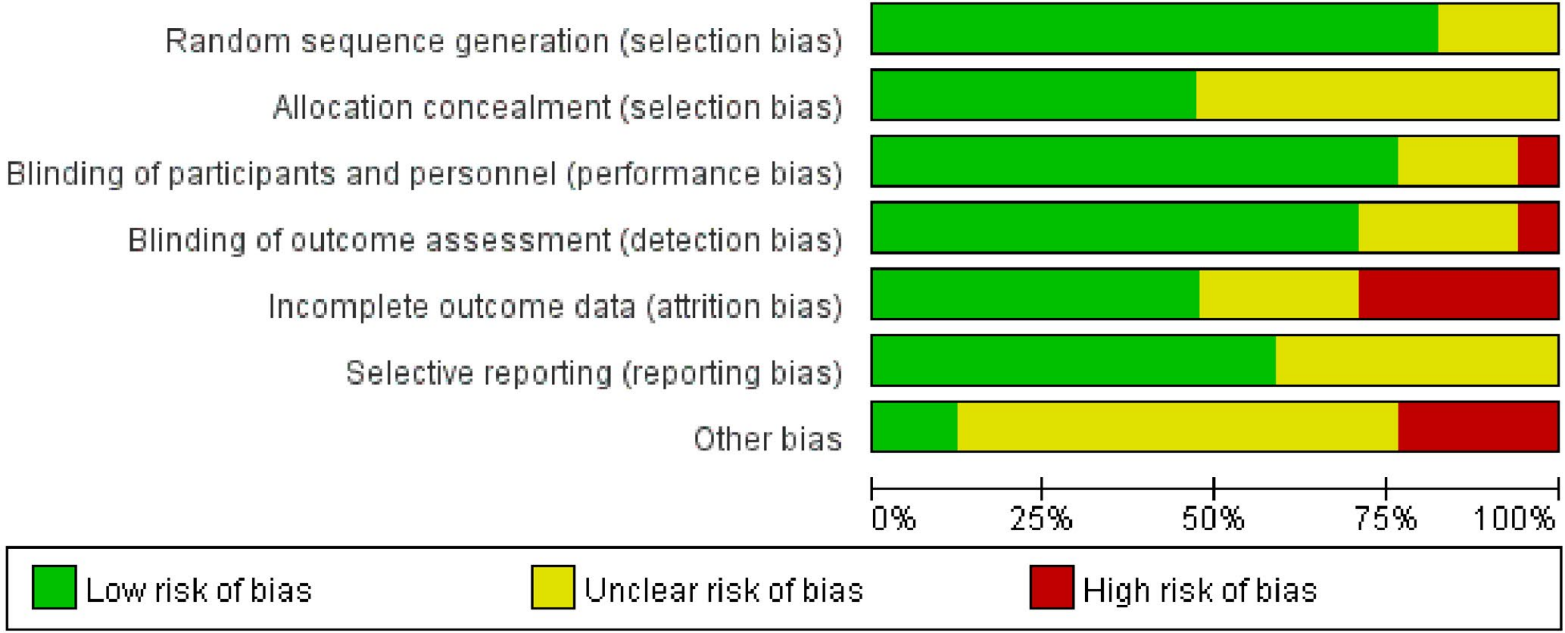
Proportions of articles that met each criterion for risk of bias across the 17 included randomized controlled trials

**FIGURE 3:**
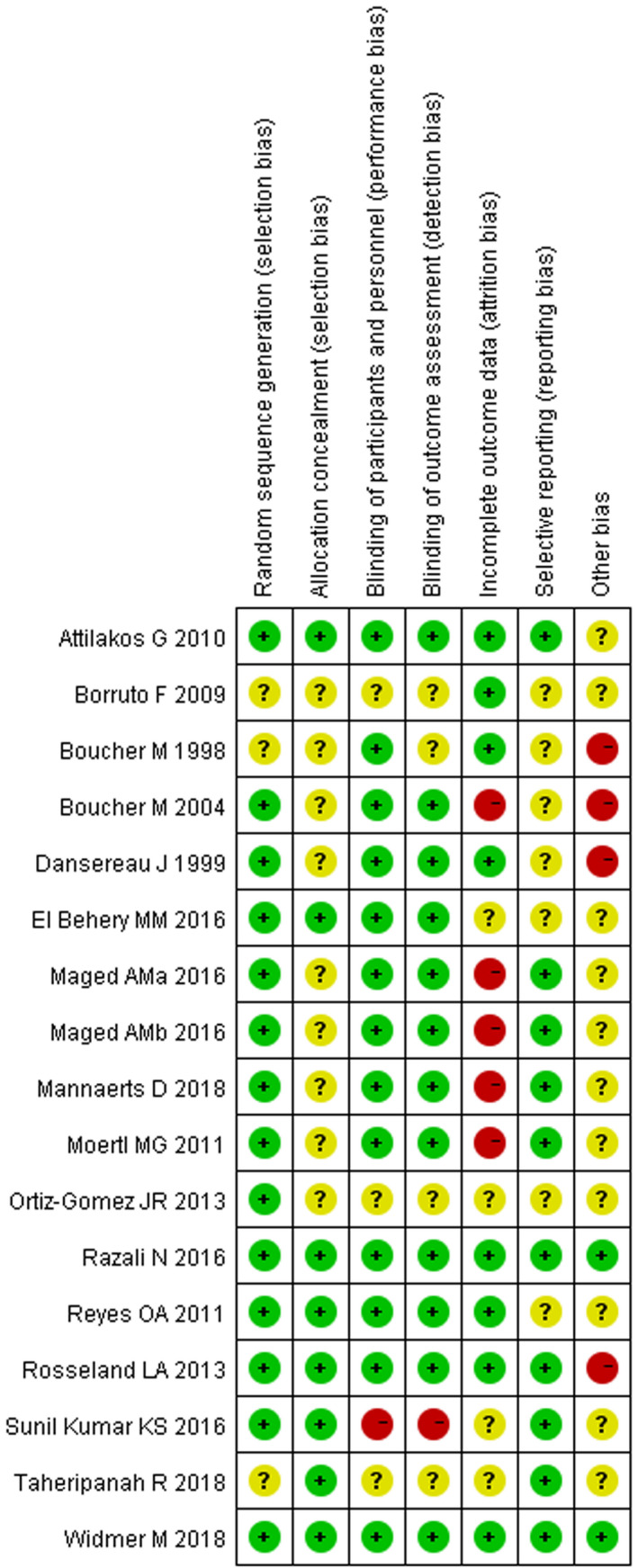
Results of the risk of bias for 17 included randomized controlled trials. Green means low risk; yellow means unclear risk; red means high risk

The quality of the included articles varied. In each of the domain, most of the articles were rated with low or unclear risk of bias. High risk of bias was mostly attributed to incomplete outcome data and other bias, such as granting by the pharmaceutical company and the failure to declare potential conflicts of interest. Meanwhile, performance and detection bias existed in one article.^15^ In general, the quality was good, with five high‐quality articles, twelve moderate quality articles, and none of the articles was classified as low quality (Figures 2, 3).

### 3.3 Quantitative analysis

**FIGURE 4:**
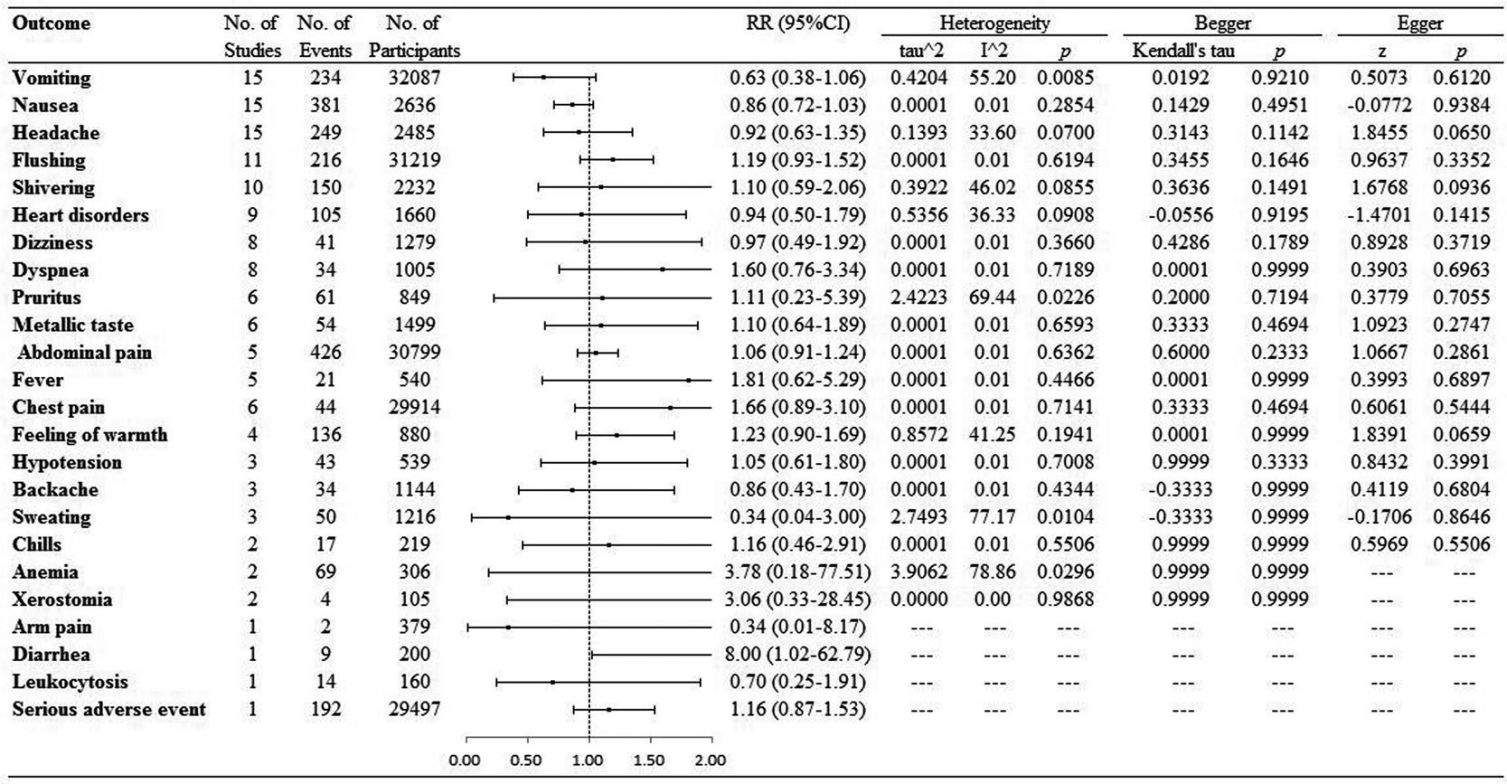
Results of side‐effects in this meta‐analysis

The use of carbetocin had a slightly higher risk of diarrhea (8.00; 1.02–62.79) compared with oxytocin intervention. In subgroup analysis, the use of carbetocin had a lower risk of vomiting in intravenously group (0.53; 0.30–0.93) and in cesarean birth women (0.51; 0.32–0.81). In addition, carbetocin use had a lower risk of vomiting (0.32; 0.18–0.55) in no fund. Except for above reporting, other side‐effects and subgroup analysis were not found different between the two interventions (Figure 4; Appendix 2).

### 3.4 Sensitivity analysis and publication bias

Sensitivity analysis by excluding a dose of 125 ug carbetocin trial^15^ showed that the results were not substantial influenced, and they were just slight changes. Funnel plots and Begger's and Egger's tests found no significant publication bias.

## 4 DISCUSSION

In this systematic review and meta‐analysis, which included 17 RCT studies covering 32,702 women, we found that vomiting, nausea, headache, and flushing are the most frequently reported side‐effects of carbetocin to PPH. The risk of vomiting decreased with carbetocin use in intravenously and cesarean birth women in the prevention of PPH after delivery. The result was based on medium‐ to high‐quality evidence.

Fewer clinical trials have focused on the side‐effects of carbetocin to prevent PPH. Mannaerts D et al^16^ have compared the adverse effects between carbetocin and oxytocin in their trial. They found the incidence of nausea was lower after carbetocin, but there was no statistical difference. A previous review, only included several studies, also showed that carbetocin is associated with fewer adverse effects.^10^ Furthermore, our study demonstrated that the administration of carbetocin in delivery is associated with the lower incidence of vomiting. We therefore suggested that carbetocin might be considered as an appropriate choose for pregnant women with vomiting intolerance for the prevention of PPH.

Studies suggested that carbetocin is superior in terms of additional uterotonics when compared with other uterotonic agent at cesarean delivery.^25^, ^28^ Meanwhile, in a prospective double‐blinded randomized study, Maged AM et al found that carbetocin is a better alternative to oxytocin in prevention of PPH after vaginal delivery in women with at least two risk factors of atonic PPH.^19^ In the subgroup analysis, we found carbetocin had a lower risk of vomiting at cesarean delivery when compared with oxytocin intervention. In addition, this analysis also demonstrated that there is a reduced risk of vomiting with carbetocin intravenously use. Considering the effects and side effects, carbetocin should be a good choice at cesarean delivery, particularly in intravenously use.

Compared with oxytocin, carbetocin has a longer half‐life, and both amplitude and frequency of contractions are prolonged when administered postpartum.^29^ Carbetocin has an efficacy and safety profile very similar to oxytocin.^30^ A Cochrane review showed that carbetocin significantly reduces the need for additional uterotonics compared to oxytocin.^10^ However, the cost of carbetocin is prohibitively expensive. The absolute cost of carbetocin is several times higher than oxytocin.^31^ Some researchers have even found there is no economic benefit with the use of carbetocin for women from the point of view of health system.^32^, ^33^

While many systematic reviews have been published on the carbetocin use, these studies mostly discuss the efficacy aspect of carbetocin in the prevention of PPH. Our study, however, is the first review amalgamating the evidence from available RCT studies with a focus on the safety aspect of carbetocin. Our review benefits from a comprehensive search strategy which captures 17 trials involving 32,072 women and 24 most common side‐effects occurring due to carbetocin. The large sample size achieved high precision results, particularly in the rare side‐effects. Moreover, the review was based on a prospective protocol which had been pre‐registered on PROSPERO registry. Furthermore, the meta‐analyses were designed carefully with strict subgroup analysis of participants and carbetocin administration characteristics. Meanwhile, sensitivity analyses demonstrated that the findings were robust.

However, some limitations in this review or in the included studies should be noted. Many of the included side‐effects were small or none in size, presenting a possibility to generate spurious associations. The time span of the included RCTs was more than 20 years, involving more than a dozen developed or developing countries and regions. Some symptoms and signs, such as flushing, feeling of warmth, and tremors, may be defined inconsistently in different trials. This may increase the risk of merging apple and orange.

## 5 CONCLUSION

The overall results of our systematic review and meta‐analysis study may raise concerns about the potential side‐effects of uterotonic agents use for preventing postpartum hemorrhage. It may help clinicians better understand the side‐effects, particularly the unanticipated side‐effects, of carbetocin use during labor and delivery. These results provide insights toward optimizing clinical decision‐making strategies, which should consider the potential benefits of using carbetocin to prevent PPH in parts of the world where a lack of cold chain transported and stored.

## CONFLICT OF INTEREST

The authors declare that they have no competing interests.

## AUTHORS’ CONTRIBUTIONS

DF and YZ participated in the design and coordination of the study. WA conceived the study and drafted the manuscript. YZ, YM, and LL searched for the studies, collected, and analyzed the data. DF participated in the design of this study and edited the manuscript. DF, SW, YZ, and YM did the data management and analyzed the data. All authors read and approved the final manuscript.

## Supporting information

Appendix S1

Appendix S2

## Data Availability

The data used to support the findings of this study are included within the article.
