# Supplementary material for: Side‐effects of carbetocin to prevent postpartum hemorrhage: A systematic review and meta‐analysis of randomized controlled trials
Source: Pharmacol Res Perspect. 2021 Mar 15;9(2):e00745. doi: 10.1002/prp2.745 (PMC7961157; doi:10.1002/prp2.745)
Supplement: Supplementary file 1 — Appendix S1 [file PRP2-9-e00745-s003.doc]

((randomized controlled trial[Publication Type]) OR (controlled clinical trial[Publication Type]) OR randomized[Title/Abstract] OR placebo[Title/Abstract] OR drug therapy [subheading] OR randomly[Title/Abstract] OR trial[Title/Abstract] OR groups[Title/Abstract]) AND ((third stage[All Fields]) AND (labor[All Fields] OR labour[All Fields]) AND Carbetocin[All Fields] AND (haemorrhage[All Fields] OR hemorrhage[All Fields]) AND postpartum[All Fields])
