# Supplementary material for: Side‐effects of carbetocin to prevent postpartum hemorrhage: A systematic review and meta‐analysis of randomized controlled trials
Source: Pharmacol Res Perspect. 2021 Mar 15;9(2):e00745. doi: 10.1002/prp2.745 (PMC7961157; doi:10.1002/prp2.745)
Supplement: Supplementary file 2 — Appendix S2 [file PRP2-9-e00745-s001.pdf]

Side-effect	Outcome		No of Studies	No of Done	No of Participants	RR (95%%CI)	R/F	Heterogeneity tau^2	Heterogeneity I^2	Heterogeneity p	Begger Kendall's tau	Begger p	Egger z	Egger p
**Vomiting**	**Overall**		15	234	32087	0.63 (0.38 to 1.06)	R	0.4204	55.20	0.0085	0.0192	0.9210	0.5073	0.6120
	**Type**													
		IV	11	161	2024	0.53 (0.30 to 0.93)	R	0.3586	48.56	0.0326	0.0909	0.7612	0.5193	0.6035
		IM	4	73	30063	1.21 (0.74 to 1.97)	F	0.7022	36.71	0.1414	0.6000	0.2511	-0.0470	0.9625
	**Dose**													
		100	14	231	31885	1.21 (0.74 to 1.97)	R	0.4151	56.41	0.0085	-0.0110	0.9999	0.0655	0.9478
		125	1	3	202	5.00 (0.24 to 102.85)								
	**Delivery**													
		VD	6	77	30222	1.26 (0.79 to 2.03)	F	0.0001	0.01	0.2937	0.1429	0.6971	0.4682	0.6396
		CS	9	157	1865	0.48 (0.27 to 0.85)	R	0.3682	53.87	0.0239	-0.1667	0.6122	-0.7759	0.4378
	**Risk**													
		None	3	18	265	0.50 (0.19 to 1.30)	F	0.0000	0.00	0.7115	0.9999	0.3333	0.8228	0.4106
		L	4	78	30136	1.09 (0.70 to 1.72)	F	0.0001	0.01	0.4120	0.0001	0.9999	-0.3572	0.7209
		H	8	138	1686	0.56 (0.22 to 1.37)	R	0.9250	70.31	0.0021	0.0001	0.9999	0.8161	0.4144
	**Drug**													
		Oxytocin	15	234	32087	0.63 (0.38 to 1.06)	R	0.4204	55.20	0.0085	0.0192	0.9210	0.5073	0.6120
	**Trial**													
		no	10	142	1831	0.51 (0.24 to 1.07)	R	0.6530	57.32	0.0045	0.3182	0.2069	1.0944	0.2738
		yes	5	92	30256	1.04 (0.69 to 1.57)	F	0.0001	0.01	0.6576	-0.2000	0.8167	-0.6395	0.5225
	**Fund**													
		Reseach	2	17	322	0.97 (0.38 to 2.48)	F	0.0001	0.01	0.4676	0.9999	0.9999	0.7264	0.4676
		Company	3	132	30533	1.06 (0.76 to 1.49)	F	0.0001	0.01	0.5555	-0.3333	0.9999	-0.9570	0.3385
		None	10	85	1232	0.32 (0.18 to 0.55)	F	0.3936	31.64	0.1178	0.4545	0.0714	2.4846	0.0130
**Nausea**	**Overall**		15	381	2636	0.86 (0.72 to 1.03)	F	0.0001	0.01	0.2854	0.1429	0.4951	-0.0772	0.9384
	**Type**													
		IV	12	361	2076	0.86 (0.71 to 1.03)	F	0.0001	0.01	0.1805	0.0606	0.8406	-0.4222	0.6729

		IM	3	20	560	0.91 (0.38 to 2.20)	F	0.0001	0.01	0.4943	0.9999	0.3333	1.0616	0.2884
	**Dose**													
		100	14	377	2436	0.86 (0.71 to 1.03)	F	0.0001	0.01	0.2258	0.0769	0.7472	-0.1291	0.8973
		125	1	4	200	1.00 (0.14 to 6.96)								
	**Delivery**													
		VD	5	25	717	1.08 (0.49 to 2.41)	F	0.0001	0.01	0.6806	0.6000	0.2333	1.4090	0.1588
		CS	10	356	1919	0.85 (0.70 to 1.02)	F	0.0001	0.01	0.1286	-0.2000	0.4843	-1.2117	0.2256
	**Risk**													
		None	3	44	265	0.98 (0.57 to 1.69)	F	0.0000	0.00	0.5115	0.9999	0.3333	1.0346	0.3008
		L	3	28	635	0.96 (0.46 to 2.01)	F	0.0001	0.01	0.4900	-0.3333	0.9999	-0.7836	0.4333
		H	9	309	1736	0.84 (0.68 to 1.02)	F	0.0438	21.89	0.1014	0.1667	0.6122	-0.3443	0.7306
	**Drug**													
		Oxytocin	15	381	2636	0.86 (0.72 to 1.03)	F	0.0000	0.00	0.2854	0.1429	0.4951	-0.0772	0.9384
	**Trial**													
		no	10	311	1825	0.84 (0.69 to 1.03)	F	0.0355	17.20	0.1087	0.1111	0.7275	-0.1964	0.8443
		yes	5	70	811	0.92 (0.59 to 1.44)	F	0.0001	0.01	0.7471	-0.2000	0.8167	-0.0931	0.9258
	**Fund**													
		Reseach	3	46	376	0.91 (0.53 to 1.55)	F	0.0001	0.01	0.7785	0.3333	0.9999	0.3895	0.6969
		Company	2	203	1036	0.93 (0.73 to 1.18)	F	0.0001	0.01	0.5009	0.9999	0.9999	0.6731	0.5009
		None	10	132	1224	0.71 (0.51 to 1.00)	F	0.1700	34.64	0.1274	0.1556	0.6007	0.7057	0.4803
**Headache**	**Overall**		15	249	2485	0.92 (0.63 to 1.35)	R	0.1393	33.60	0.0700	0.3143	0.1142	1.8455	0.0650
	**Type**													
		IV	13	225	2125	0.94 (0.62 to 1.43)	R	0.1519	35.74	0.0780	0.3333	0.1289	1.6508	0.0988
		IM	2	24	360	0.98 (0.21 to 4.57)	R	0.8197	64.24	0.0945	0.9999	0.9999		
	**Dose**													
		100	15	249	2485	0.92 (0.63 to 1.35)	R	0.1393	33.60	0.0700	0.3143	0.1142	1.8455	0.0650
	**Delivery**													
		VD	4	30	519	0.96 (0.45 to 2.07)	F	0.8146	48.16	0.1339	0.3333	0.7500	2.1069	0.0351
		CS	11	219	1966	0.88 (0.59 to 1.32)	R	0.1159	33.19	0.0783	0.2727	0.2830	1.0896	0.2759

	**Risk**													
		None	3	24	267	2.29 (0.93 to 5.59)	F	0.2248	18.71	0.3848	0.3333	0.9999	0.9467	0.3438
		L	1	4	377	1.01 (0.14 to 7.06)								
		H	11	221	1841	0.79 (0.55 to 1.14)	R	0.0791	25.73	0.0989	0.2000	0.4454	1.2874	0.1980
	**Drug**													
		Oxytocin	14	246	2433	0.90 (0.62 to 1.30)	R	0.1224	32.15	0.0697	0.3187	0.1268	1.5854	0.1126
		Placebo	1	3	52	5.00 (0.25 to 99.16)								
	**Trial**													
		no	9	216	1627	0.98 (0.66 to 1.47)	R	0.1156	38.53	0.0623	0.3889	0.1802	2.1788	0.0293
		yes	6	33	858	0.64 (0.29 to 1.41)	F	0.4506	30.07	0.2365	0.7333	0.0556	2.2849	0.0223
	**Fund**													
		Reseach	3	21	378	0.41 (0.14 to 1.20)	F	1.1276	51.17	0.1298	0.9999	0.3333	1.7787	0.0753
		Company	4	101	1139	1.07 (0.74 to 1.55)	F	0.0001	0.01	0.7347	0.3333	0.7500	0.3238	0.7461
		None	8	127	968	1.13 (0.59 to 2.16)	R	0.4113	58.21	0.0516	0.5000	0.1087	2.5601	0.0105
**Flushing**	**Overall**		11	216	31219	1.19 (0.93 to 1.52)	F	0.0001	0.01	0.6194	0.3455	0.1646	0.9637	0.3352
	**Type**													
		IV	9	205	1520	1.18 (0.92 to 1.52)	F	0.0001	0.01	0.4570	0.2778	0.3585	0.8285	0.4074
		IM	2	11	29699	1.42 (0.42 to 4.78)	F	0.0001	0.01	0.6185	0.9999	0.9999	0.4979	0.6185
	**Dose**													
		100	11	216	31219	1.19 (0.93 to 1.52)	F	0.0001	0.01	0.6194	0.3455	0.1646	0.9637	0.3352
	**Delivery**													
		VD	4	15	29858	1.70 (0.59 to 4.95)	F	0.0001	0.01	0.8894	0.0001	0.9999	0.7918	0.4285
		CS	7	201	1361	1.16 (0.90 to 1.50)	F	0.0001	0.01	0.3205	-0.0476	0.9999	0.4443	0.6568
	**Risk**													
		None	0											
		L	3	22	29932	1.37 (0.59 to 3.18)	F	0.0001	0.01	0.9692	0.9999	0.3333	0.2365	0.8130
		H	8	194	1287	1.17 (0.91 to 1.52)	F	0.0001	0.01	0.3406	0.2143	0.5484	0.8885	0.3743
	**Drug**													
		Oxytocin	10	214	31167	1.20 (0.93 to 1.53)	F	0.0000	0.00	0.5883	0.4222	0.1083	1.2916	0.1965

	Placebo	1	2	52	0.33 (0.01 to 7.81)								
**Trial**													
	no	4	180	1024	1.18 (0.91 to 1.54)	F	1.1182	47.93	0.1307	0.0001	0.9999	1.9804	0.0477
	yes	7	36	30195	1.20 (0.62 to 2.35)	F	0.0001	0.01	0.8731	0.1429	0.7726	-0.7862	0.4318
**Fund**													
	Reseach	2	9	158	1.52 (0.42 to 5.49)	F	0.0001	0.01	0.6472	0.9999	0.9999	0.4577	0.6472
	Company	5	183	30638	1.12 (0.87 to 1.45)	F	0.0001	0.01	0.7577	-0.6000	0.2333	-0.8268	0.4083
	None	4	24	423	3.25 (0.99 to 10.66)	F	0.2426	12.31	0.4057	0.0001	0.9999	1.0144	0.3104
